# Respiratory motion effects and plan robustness for lattice radiation therapy

**DOI:** 10.3389/fonc.2026.1731981

**Published:** 2026-02-25

**Authors:** Rachael M. Martin-Paulpeter, Peter A. Balter, Luis A. Perles, Ethan B. Ludmir, Joshua S. Niedzielski

**Affiliations:** 1 Department of Radiation Physics, The University of Texas MD Anderson Cancer Center, Houston, TX, United States; 2 Department of Gastrointestinal Radiation Oncology, The University of Texas MD Anderson Cancer Center, Houston, TX, United States

**Keywords:** lattice therapy, liver, motion management, radiation therapy, respiratory motion, SFRT

## Abstract

**Introduction:**

Lattice radiation therapy (LRT), a form of spatially fractionated radiation therapy (SFRT), has shown promise in treating bulky tumors. It consists of hot and cold spots within the tumor, and the ratio between these doses is thought to be important in clinical outcomes. Respiratory motion is expected to degrade these peak-to-valley dose ratios (PVDRs) but has largely been ignored in LRT literature and clinical practice. This work aims to quantify the response of LRT dose distributions to motion to better inform motion management decisions and improve clinical outcomes.

**Methods:**

Respiratory motion of peak-to-peak amplitudes from 0.3 to 2 cm was simulated in 1 and 1.5 cm sphere lattice plans in a uniform phantom using dose perturbations. A similar analysis was repeated with retrospective patient plans to understand the effect of anatomical variation in motion response. Finally, deformed and rigid dose transformations were compared to understand the effect of tumor deformation on motion response.

**Results:**

Hot spheres (VTVH) lost coverage with increasing motion, while cold spheres (VTVL) experienced either an increase (anterior–posterior motion) or a decrease (superior–inferior motion). The ratio of the two (VTVH/VTVL D95%) decreased with increasing motion. Patient data results generally agreed with phantom results; 1.5 cm sphere plans were less sensitive to motion than 1 cm sphere plans, with statistical differences between the two responses. Mean (standard deviation) percent changes from no motion to 1 cm superior–inferior motion for patient plans are as follows with phantom values listed after: VTVH D80% change was −13.8 (1.2), −12.7 for 1.5 cm spheres and −15.8 (1.8), −13.8 for 1 cm spheres; and VTVH/VTVL D95% change was −7.1 (4.0), −15.0 and −13.0 (3.5), −12.0, respectively. Generally, no significant difference was observed between deformed and perturbed dose distributions.

**Discussion:**

Large tumor motion can degrade LRT dose distributions, which could lead to less effective treatments. Suggested cutoffs of 1 cm motion for 1.5 cm sphere plans and 0.5 cm motion for 1 cm sphere plans are proposed above which motion limiting strategies should be applied. Caution should be applied for tumors with large deformations, as these may change the dose distributions in unexpected ways.

## 1 Introduction

Spatially fractionated radiation therapy (SFRT) has been in use in the form of grid therapy since the orthovoltage era to spare normal tissues such as the skin (1). In recent years, VMAT-based lattice radiation therapy (LRT) was introduced, which can extend the benefits of SFRT to deep-seated tumors (2–9). Since then, lattice and grid therapy have shown promising initial results, particularly in bulky tumors (10–21). Lattice therapy is characterized by a lattice of hot spots and cold spots with sharp dose gradients between the two. While the specific qualities of an ideal lattice plan are still under investigation, the peak-to-valley dose ratio (PVDR) and related dose metrics, as well as gross tumor volume (GTV) mean, equivalent uniform dose (EUD), and volumetric dose metrics, are thought to be important (22–24).

Several common LRT targets, such as the liver and thorax, are subject to respiratory motion. Respiratory motion is expected to decrease the PVDR by smearing out the dose distribution and thereby increasing cold spot dose and decreasing coverage of the hot spots. Naqvi et al. (25) modeled motion for SFRT with a physical grid using Monte Carlo-based motion models of a phantom. They found noticeable degradation of the GRID pattern beyond a few millimeters of motion, with effects being accentuated for tighter GRID spacings. While similar results are to be expected with a VMAT-based lattice, there is enough difference in the underlying dose distributions that the exact extent of the effect of motion cannot be directly extrapolated from these results. Beyond Naqvi’s study, the effect of motion on SFRT dose distributions has been largely ignored in the literature, and clinical practice has often opted not to use motion-limiting strategies such as breath hold or compression belt. Appropriately applying motion management strategies can improve outcomes for patients receiving LRT to the abdomen or thorax. Additionally, as SFRT clinical trials gain momentum, having a clear understanding of how motion impacts lattice dose distributions will improve the interpretation of outcome data for abdominal and thoracic tumors.

Due to the high complexity of lattice plans and generally large tumor sizes, treatment times can be quite long, often over 10 min of beam-on time even with flattening filter-free (FFF) beams. These long delivery times make LRT treatments with breath-hold motion management more challenging for patients and increase the chance of gross patient movement during breath-hold or free-breathing gated treatments. Understanding how much motion can be tolerated with free breathing without appreciably degrading the dose distribution allows for a more informed decision on when free breathing would be acceptable or if a more aggressive motion management strategy is warranted.

The aim of this study is to address the gap in knowledge regarding lattice motion effects by systematically evaluating the impact of respiratory motion on lattice dose distributions and dose metrics. The study will evaluate two commonly used lattice patterns and compare their sensitivity to motion. The effect of anatomical differences and deformation will be explored to further understand the complexities of motion response. This study focuses on liver tumors, as they are a common lattice target affected by motion. While bulky lung tumors are also treated with lattice, the large tumor size common for lattice treatments is likely to impact lung function such that respiratory motion is minimal.

## 2 Methods

### 2.1 Lattice treatment planning

**Figure 1:**
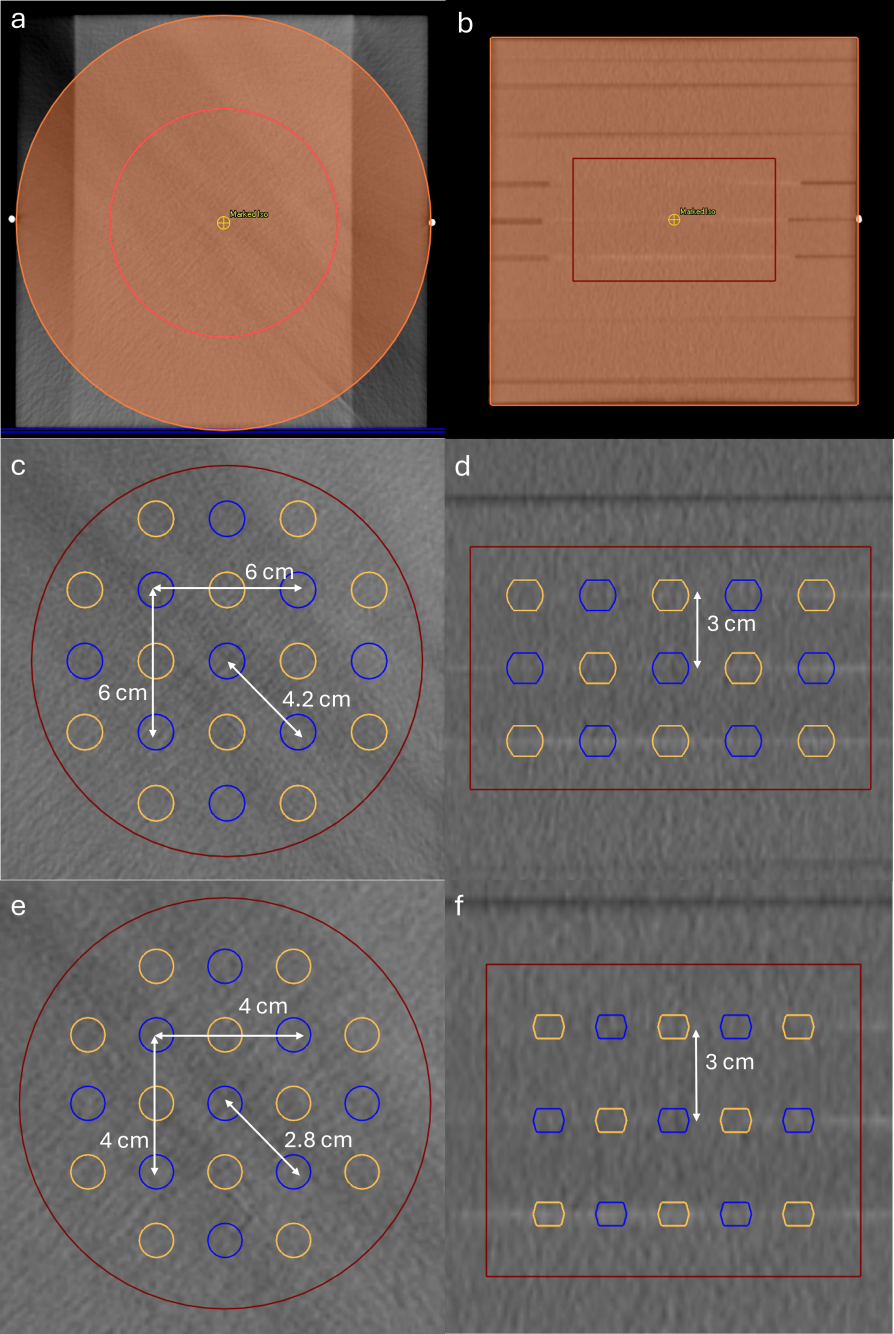
Axial **(A)** and coronal **(B)** view of the phantom. The material of the orange cylinder was overridden by water and outside the cylinder by air. GTV is shown in red. Lattice patterns for 1.5 cm spheres (middle) and 1 cm spheres (bottom) for phantom plans. VTVH is shown in blue, VTVL in orange, and GTV in red. **(C, E)** An axial slice and **(D, F)** a coronal slice.

All treatment planning was done using RayStation version 11B (RaySearch Laboratories, Stockholm, Sweden). The lattice plans created for this study consist of hot spheres referred to collectively as vertex volume-high (VTVH) and cold spheres or vertex volume-low (VTVL). This general approach was adopted from Duriseti et al. (3) and is also seen in Burns et al. (26) While there are other valid strategies for obtaining a lattice pattern, this method was chosen, as it more clearly defines the valley region. PVDR, as a concept, is thought to be important for SFRT. However, it is difficult to define consistently with VMAT-based lattice therapy since line profiles can be drawn in any number of directions between hot spheres, resulting in different PVDRs. Having clearly defined peak and valley regions allows for a consistent surrogate for PVDR, improving plan consistency and more quantitative evaluation of the effect of respiratory motion. The hot spheres are arranged into one of two commonly used lattice patterns as shown in **Figure 1**. The first consists of 1 cm diameter spheres that are spaced 4 cm apart in the right–left (RL) and anterior–posterior (AP) directions and 3 cm in the superior–inferior (SI) direction. Each row and slice of spheres is offset from the others to form a diamond-shaped lattice pattern. The second pattern is similar but with 1.5 cm diameter spheres spaced 6 cm apart in the RL and AP directions and 3 cm apart in the SI direction.

**Figure 2:**
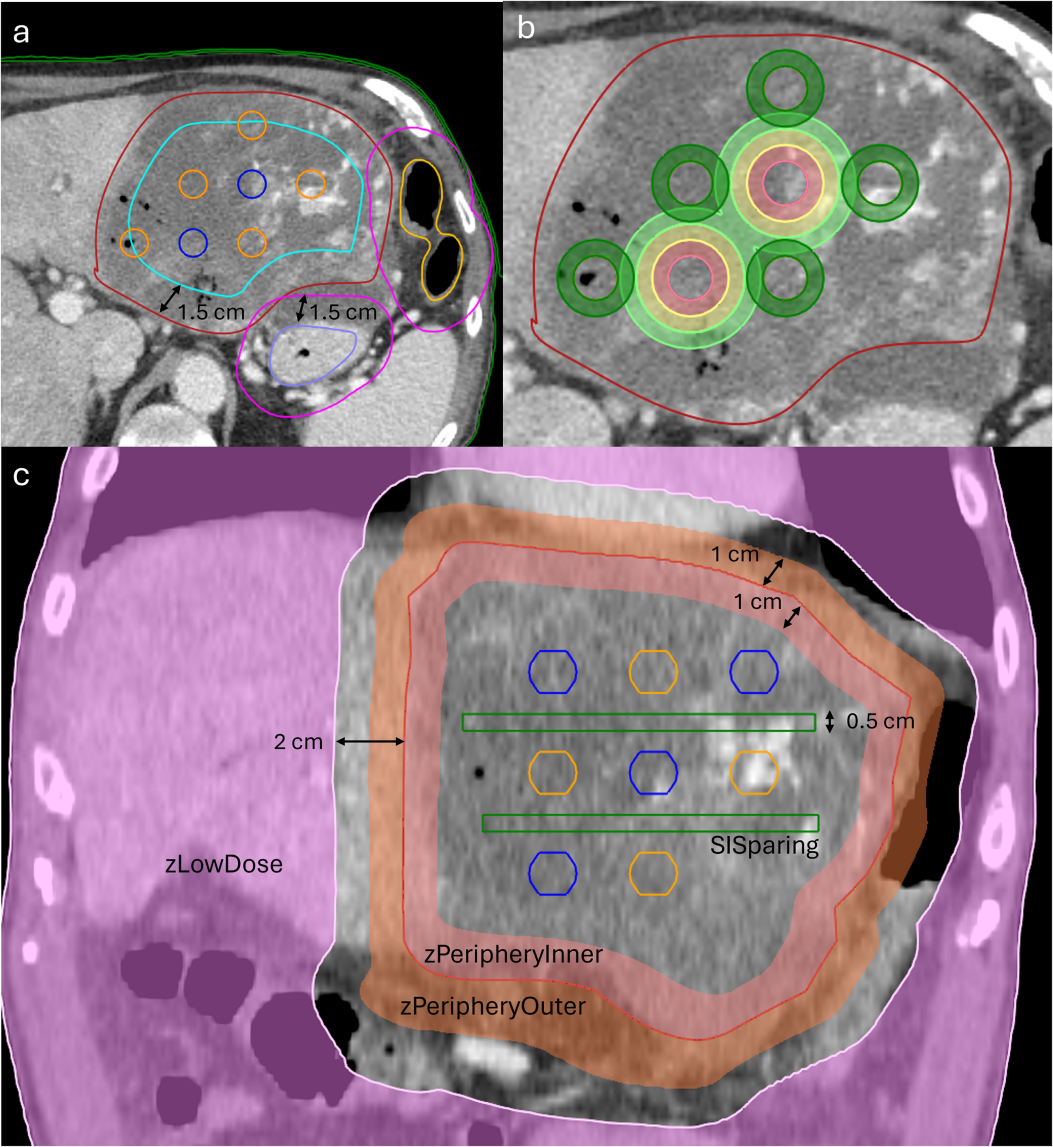
Target and optimization structures used in LRT treatment planning. The GTV is in dark red, VTVH in blue, and VTVL in orange. LRT_PRV (pink) and LRT_GTV (cyan) are shown in **(A)** with margins appropriate for a 1.5-cm sphere plan (1 cm margins used for a 1-cm sphere plan). The LRT_GTV is defined as a contraction of the GTV minus the LRT_PRV. VTVH must remain completely within LRT_GTV, while VTVL can extend outside LRT_GTV but must remain completely in the GTV. Ring contours are shown in **(B)** with three 5 mm (2 mm for 1 cm sphere plans) thick rings for VTVH and one for VTVL (same thicknesses). **(C)** zLowDose (pink), which is defined as external minus (GTV plus 2 cm margin); zPeripheryOuter (orange), which is a 1-cm expansion ring on the GTV; and zPeripheryInner (salmon), which is a 1-cm contraction ring on the GTV; 0.5-cm-thick SISparing boxes (green) are placed between spheres in the SI direction and just encompass the LRT_GTV in the AP and RL directions.

Hot spheres are constrained to be completely within a contracted version of the gross tumor volume (GTV), labeled as LRT_GTV, which also excludes a planning organ at risk volume (PRV). A GTV contraction and PRV margin equal to the diameter of the spheres is used for each pattern. Cold spheres are allowed to extend outside of the LRT_GTV so long as they are within the GTV. The LRT_GTV was created, and spheres were placed using an in-house automated script run through RayStation that allows the user to select the desired lattice pattern and organs at risk (OARs) to include within the PRV. In addition to objectives placed on VTVH and VTVL, various planning structures, including rings around VTVH and VTVL, peripheral rings for the GTV, and rectangular structures between slices (SISparing), were used to achieve the desired lattice pattern dose distribution. SISparing OARs were created by placing a rectangle with a height of 0.5 cm and a width just large enough to encompass the LRT_GTV between spheres in the SI direction and are used to enhance the PVDR in the SI direction. **Figure 2** shows the different OARs used in LRT planning, and **Supplementary Figure 1** gives examples of optimization objectives and constraints used in planning.

Depending on the complexity of the plan, between 4 and 6 co-planar arcs are used with different collimator angles represented (typically 0, 90, and 45). A 1.5-mm dose grid is used to sufficiently capture the high-dose gradients inherent in these plans. Dose is prescribed as 20 Gy in 1 fraction to 80% of VTVH. Dose was calculated with RayStation’s Collapsed Cone Version 5.7.

### 2.2 Phantom motion analysis

As part of an end-to-end test for clinical commissioning, a cubic (30 cm × 30 cm × 30 cm) solid water phantom was scanned with a Philips Big Bore CT scanner (Philips, Andover, MA, USA). A cylindrical phantom was imitated using material overrides to water within a 30-cm-diameter and 30-cm-long cylinder and air outside of it. Lattice plans using 1 and 1.5 cm spheres were created with this phantom using a centrally located sphere as outlined in Section 2.1. To minimize the confounding effects of the number and placement of hot spheres, the same scaled pattern with spheres placed in three axial planes and the total number of spheres (17) was used for each lattice spacing. GTVs consisted of cylinders with heights of 10 cm and diameters of 12 and 16.5 cm (1,130 and 2,135 cm^3^) for 1 and 1.5 cm sphere plans, respectively. The isocenter was placed at the center of the phantom.

**Figure 3:**
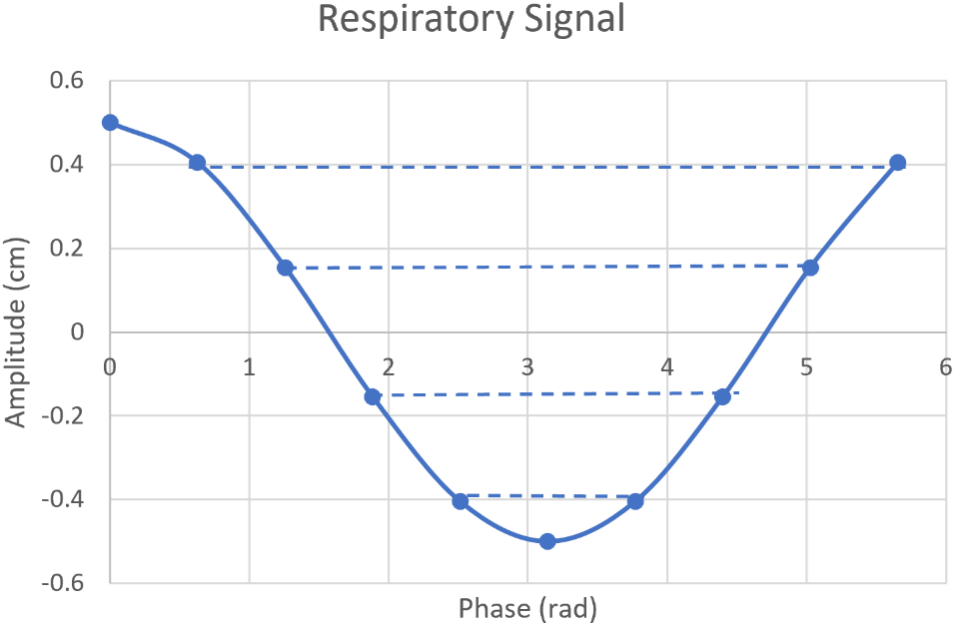
Example of 1 cm peak-to-peak respiratory signal sampled at 10 locations. Dotted lines indicate duplicated values.

The dose perturbation tool in RayStation was used to recalculate the dose in different simulated phantom positions. This tool shifts and rotates the patient by a specified amount before recalculating the dose. Translation-only perturbations with this tool are equivalent to moving the isocenter and recalculating the dose, and are the only type of perturbation used in this study. For the remainder of this paper, doses calculated using this perturbation tool are referred to as perturbed doses to distinguish them from deformed doses. To mimic respiratory motion, cosine waves were sampled at 10 evenly spaced points along the curve, which resulted in six unique values per amplitude. Peak-to-peak amplitudes of 0.3, 0.5, 1.0, 1.5, and 2.0 cm were sampled. An example of this sampling for 1 cm motion is shown in **Figure 3**. The sampled values were used to perturb the dose in the SI, AP, and AP and SI directions combined. For the combined direction, a vector distance equal to the sampled values was used, and AP and SI each had the same contribution to the overall motion. The LR direction was not explicitly studied due to the symmetry of the lattice pattern and the cylindrical phantom in the AP and LR directions. For each amplitude and direction, a weighted sum of all perturbations was computed such that the combined weight equals 1. These summed dose distributions represent the effect of respiratory motion on the dose distribution for the given peak-to-peak amplitude and direction.

Dose metrics were recorded for each summed and unperturbed dose distribution. These included average doses of VTVH and VTVL, dose at percent volume for VTVH and VTVL (D5%, D80%, D90%, and D95%), and the ratio of VTVH to VTVL for each dose at percent volume (VTVH/VTVL D80%, D90%, and D95%, and VTVH/VTVL D5/D95%) to act as a surrogate for PVDR. D95% was additionally calculated for the SISparing contour, as it is a measure of dose fall off between axial planes, and VTVH/SISparing D5/D95% was calculated as a PVDR surrogate focusing on the SI direction. Additionally, GTV metrics were recorded as proposed by Zhang et al. (22) including D5%, D10%, D20%, D50%, D90%, and D90/D10% (surrogate for 1/PVDR). The effect on these dose metrics of changing motion amplitude and direction was studied for each lattice pattern.

### 2.3 Patient anatomical variation analysis

A retrospective study with patient lattice plans was performed to understand the effect of anatomical variation including GTV shape on the results from the phantom study using rigid motion. This study population consisted of 15 patients with tumors in the liver, ranging in volume from 552 to 2,011 cm^3^. **Table 1** includes information on the patients included in this study and the lattice treatment plans created to put the results of this and the following analysis in context. Simulations consisted of either 4–6 breath-hold (BH) scans (12 patients) or a 4D computed tomography (4DCT) scan (3 patients). For patients treated with BH, RPM was used to track their breathing. Initially, they were instructed to take a comfortable breath in and hold several times without visual feedback, and a 0.5-cm gating window was set based on their natural BH. For subsequent BHs and during treatment, patients had visual feedback of their breathing and gating target to aid in consistent BHs. After 1–2 non-contrast BHs or before the 4DCT, patients were injected with iodinated contrast, with 3–4 BH images taken back-to-back starting at 30 s or 4DCT starting as soon as possible after injection. An internal gross tumor volume (IGTV) is contoured using the tumor position across all BH images or 4DCT phases. For each patient, lattice plans with 1 and 1.5 cm spheres were created using the method described in Section 2.1. To keep the treated area consistent between 1 and 1.5 cm sphere plans, a 1.5-cm PRV and GTV contraction margin was used for large tumors (>1,000 cm^3^) and 1 cm margins for smaller tumors for both lattice patterns.

**Table 1:** Information on patients and lattice treatment plans used in retrospective analyses including GTV volume, the largest tumor motion between BH or 4DCT phase images, the number of spheres in the treatment plan, and the number of axial planes where the centers of spheres were placed.

		Rigid tumor motion (cm)			Number of spheres		Axial slices with spheres	
Patient #	GTV volume (cm³)	RL	SI	AP	1 cm	1.5 cm	1 cm	1.5 cm
1	2,011	0.2	0.1	0.2	18	11	4	3
2	1,686	0.2	0.6	0.4	21	9	5	4
3	1,271	0.3	0.5	0.3	14	5	3	3
4	897	NA	NA	NA	10	6	3	3
5	733	0.1	0.2	0.1	9	3	3	2
6	552	0.1	0.4	0.1	4	3	2	2
7	1,938	0.1	0.3	0.3	22	9	5	5
8	1,872	0.1	0.7	0.3	24	11	3	3
9	1,584	0.0	0.2	0.1	20	6	3	3
10	1,358	0.6	1.8	1.1	17	7	3	3
11	1,196	0.1	0.3	0.1	17	6	4	3
12	1,066	0.0	0.4	0.2	9	4	3	3
13	993	0.2	0.2	0.1	12	5	3	3
14	786	0.1	0.4	0.1	14	6	3	3
15	660	0.2	0.8	0.2	5	3	2	2

Patient 4 did not have available 4DCT phase images, so tumor motion was not reported and this patient was excluded from the target deformation analysis described in Section 2.4.

A subsample of the simulated respiratory signals used for the phantom study was used for the anatomical variation study (0.5 and 1.0 cm amplitudes in the AP and SI directions). Doses were rigidly perturbed and summed as in the phantom study for each amplitude, direction, and sphere size, and the previously mentioned dose metrics were recorded. Percent changes in dose metrics relative to no motion were compared to phantom results and also between the different sphere sizes using paired Wilcoxon signed-rank tests. Patient values were compared to the corresponding phantom value with the same sphere size and motion amplitude and direction using a *t*-test (for parametric samples) or Wilcoxon signed-rank test (for non-parametric samples).

### 2.4 Target deformation analysis

The phantom and patient studies mentioned assume that the anatomy moves rigidly during respiration. However, for large targets, this assumption is not always valid, especially near the periphery of the liver (27). An additional analysis using the patient data in Section 2.3 was completed to better understand more realistic motion effects on liver tumors due to deformation and to determine how accurate rigid motion is for predicting motion effects.

For patients treated under breath hold, all BH CT images were rigidly and deformably registered to the planning BH image in RayStation using the liver and GTV contours as focus structures. Registrations were evaluated visually for accuracy, and rigid registrations were adjusted manually as needed. The rigid registration shifts were used as input into the dose perturbation function, and a weighted sum of all BH-determined shifts was computed. The resulting dose distribution is referred to as the perturbed dose for the remainder of this paper.

For the deformation evaluation, the dose distribution was recalculated on each BH image, then deformed back onto the planning CT using the deformable registration between the two images. Again, a weighted sum of all deformed dose distributions was computed, and the resulting dose is referred to as the deformed dose for the remainder of this paper.

For patients treated with free breathing, each phase of the 4DCT was rigidly and deformably registered to the 50% phase in a similar way to the BH CT analysis. Perturbed and deformed dose distributions were calculated on the 50% phase, similar to the BH analysis, and doses were compared to the planning dose distribution recalculated on the 50% phase. One patient did not have phase images available and so was excluded from this portion of the study. For all patients, the dose metrics described in Section 2.2 were compared between the perturbed and deformed plans using paired Wilcoxon signed-rank tests.

## 3 Results

### 3.1 Phantom rigid motion analysis

**Figure 4:**
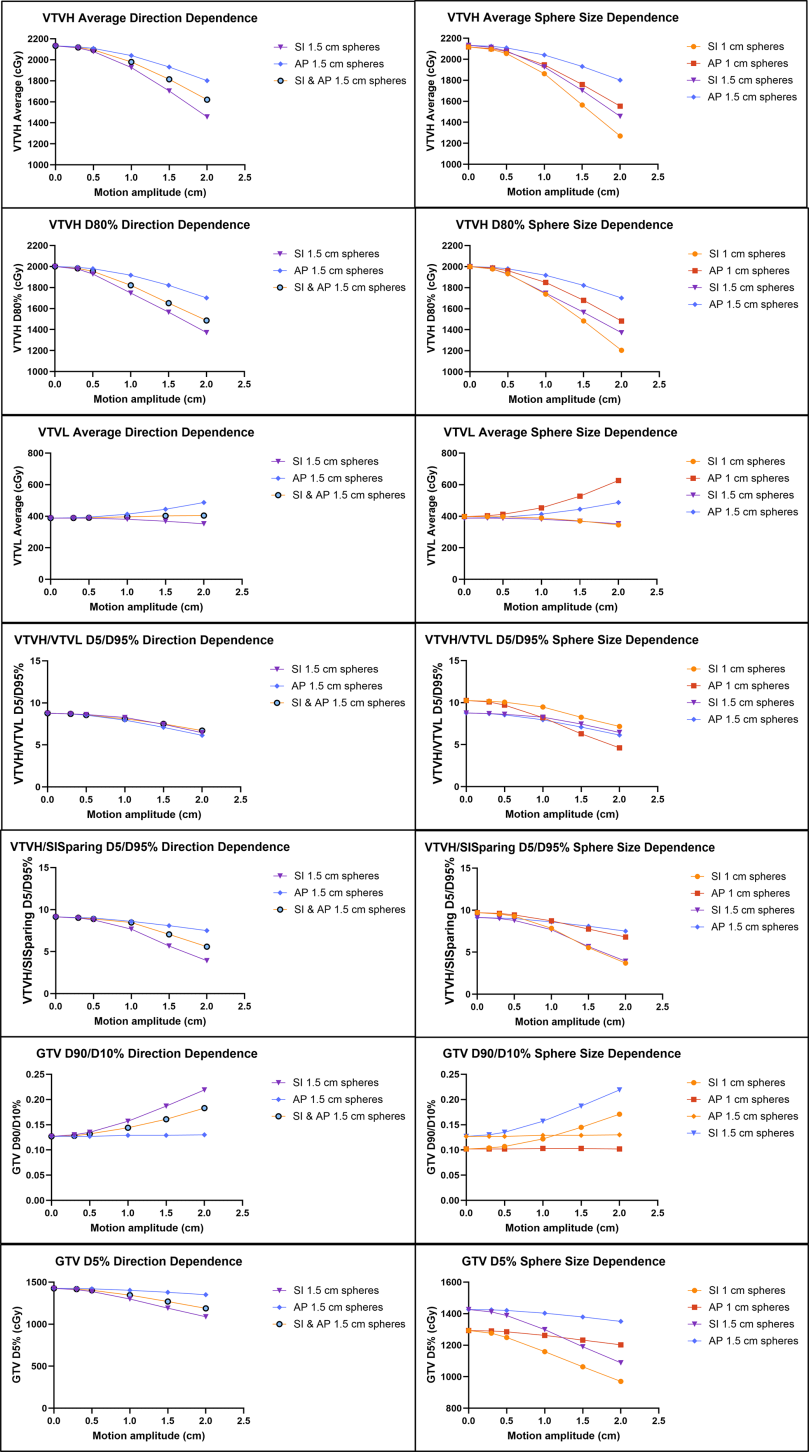
Effect of simulated respiratory motion on lattice-related dose metrics. The left graphs focus on the difference in motion response of motion direction. The right graphs focus on sphere size dependence.

Key results for the phantom summary are summarized in **Figure 4**, as well as additional metrics in **Supplementary Figure 2**, with the left graphs focusing on the impact of changing the direction of motion and the right graphs on the impact of changing the lattice pattern. Generally, dose metrics for 1 cm sphere size plans changed more rapidly than those for 1.5 cm. Hot spot coverage as measured by VTVH average dose and VTVH D80% as well as GTV D5% remained relatively unchanged up to approximately 0.5 cm of motion and then began to drop off. The drop-off was more rapid for 1 cm compared to 1.5 cm spheres and for SI motion compared to AP motion.

Cold spot dose, as quantified by VTVL average dose and VTVL D5%, was similarly minimally impacted up to 0.5 cm and changed faster for 1 cm spheres. Motion direction affected these metrics in different ways. SI motion tended to decrease the dose to the cold spots, while AP motion tended to increase it. The combined AP and SI motion had little effect on the low-dose spheres, as the effects of AP and SI motion essentially cancelled each other out.

PVDR surrogates (VTVH/VTVL D80%, 90%, and 95%) also decreased with increasing motion. GTV D90/D10% as an inverse of PVDR surrogate increased with increasing motion. The 1-cm sphere plan was able to achieve a better PVDR without motion, but the gap between the two plans decreased with increasing motion. Motion direction had less of an impact on PVDR than it did on dose to either hot or cold spots individually. The larger decrease in dose in the hot spheres with SI motion was compensated for by a decrease rather than an increase in dose to the low-dose spheres as seen with AP motion.

**Figure 5:**
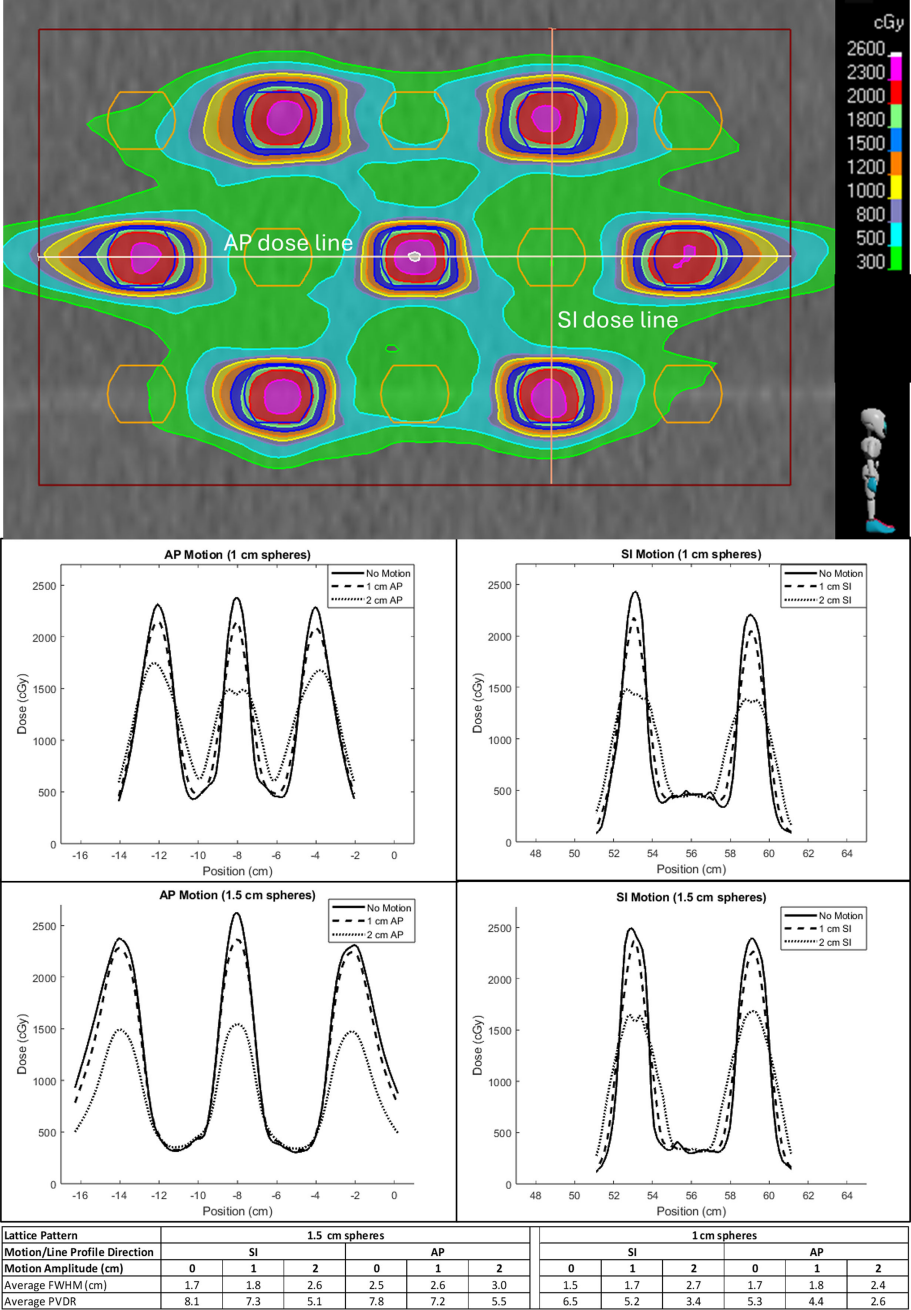
AP and SI dose line profiles for 1 and 1.5 cm sphere phantom plans. The top shows the placement of the dose line profiles on a sagittal plane through the isocenter of the phantom plan with GTV shown in red. The middle shows the effect of increasing motion (no motion solid, 1 cm dashed, and 2 cm dotted) for AP (left) and SI (right) line profiles for 1 cm (top) and 1.5 cm (bottom) sphere plans. The bottom shows a table of average full-width half maximum (FWHM) and peak-to-valley dose ratio (PVDR) for all dose lines displayed.

**Figure 5** shows the effect of changing motion amplitude and direction on dose line profiles. Line profiles were drawn along the length of the GTV in the SI and AP directions as shown in **Figure 5**. Average FWHM and PVDR across all peaks and valleys were calculated for each dose line profile shown in these graphs and displayed at the bottom of **Figure 5**.

### 3.2 Patient anatomical variation analysis

On average, the patient results were similar to those from the phantom study for the same sphere size and motion direction and amplitude. **Table 2** displays the average (standard deviation) percent changes in dose metrics from no motion alongside the values obtained in the phantom study. Significance of statistical test comparing phantom to patient percent differences from no motion showed mixed results and are displayed in **Table 2**. However, the differences between the phantom and average patient values were generally below 5 percentage points (exceptions bolded in **Table 2**), indicating that the differences are likely not notable clinically. The most notable of these exceptions were VTVH/VTVL D95%, and GTV D90/10% which somewhat overestimated in the phantom compared to patient data. GTV metrics showed more variation, likely due to the irregularly shaped GTVs of patients compared to the cylindrical shape of phantom GTVs. GTV metrics also have the limitation that they are sensitive to the different margins used between 1 and 1.5 cm plans. Generally, changes to VTVH and VTVL had less variation (smaller standard deviation) between patients than PVDR surrogates.

**Table 2:** Average (standard deviation) percent difference in dose metrics from no motion plan for different sphere sizes, motion directions, and motion amounts.

Lattice pattern	1.5 cm spheres			
Motion direction	SI		AP	
Motion amplitude (cm)	0.5	1	0.5	1
VTVH 80%	-4.0 (0.8), -3.6 (ns)	-13.8 (1.2), -12.7 (**)	-1.9 (0.4), -1.1 (****)	-6.4 (1.0), -4.2 (****)
VTVH Average	-2.9 (0.7), -2.5 (**)	-10.3 (0.8), -9.7 (**)	-1.9 (0.8), -1.1 (****)	-6.2 (1.0), -4.3 (****)
VTVL Average	-1.1 (0.4), -0.3 (****)	-4.6 (1.4), -1.8 (****)	1.3 (0.5), 1.5 (ns)	5.0 (1.4), 6.4 (**)
VTVL D5%	-1.1 (0.4), -1.1 (ns)	-3.1 (1.5), -3.3 (ns)	1.2 (0.7), 1.3 (ns)	5.0 (1.7), 5.5 (ns)
VTVH/VTVL D80%	-3.0 (1.3), -3.6 (ns)	-9.6 (2.5), -12.1 (**)	-3.0 (0.8), -2.9 (ns)	-10.1 (2.0), -10.3 (ns)
VTVH/VTVL D90%	-4.3 (2.9), -5.0 (ns)	-11.6 (3.2), -13.9 (*)	-3.6 (2.7), -2.3 (ns)	-9.8 (3.5), -9.3 (ns)
VTVH/VTVL D5/D95%	-0.9 (1.2), -1.7 (*)	-1.7 (2.8), -5.7 (****)	-2.5 (1.2), -2.6 (ns)	-8.4 (2.3), -9.4 (*)
VTVH/SISparing D5/D95%	-4.5 (0.7), -3.9 (**)	-18.1 (2.4), -16.0 (**)	-1.7 (0.6), -1.6 (ns)	-6.7 (3.0), -5.8 (ns)
VTVH/VTVL D95%	-4.7 (2.2), -5.6 (ns)	-7.1 (4.0), -15.0 (****)	-3.2 (2.3), -2.6 (ns)	-9.1 (2.8), -8.7 (ns)
GTV D5%	-2.6 (0.5), -2.7 (ns)	-7.9 (0.9), -8.9 (***)	-0.2 (0.4), -0.4 (ns)	-0.4 (0.8), -1.6 (****)
GTV D10%	-2.2 (0.2), -2.8 (****)	-6.8 (0.7), -7.7 (***)	0.0 (0.2), -0.4 (***)	0.1 (0.3), -0.8 (****)
GTV D20%	-1.5 (0.4), -0.9 (****)	-4.3 (1.0), -2.3 (****)	0.1 (0.2), 0.1 (ns)	0.6 (0.4), 0.5 (ns)
GTV D50%	5.2 (1.3), 2.6 (****)	15.7 (4.0), 7.9 (****)	0.4 (0.3), 0.2 (ns)	0.7 (0.4), 1.4 (****)
GTV D90%	1.9 (2.2), 3.5 (*)	8.1 (7.7), 14.2 (**)	-0.3 (0.9), 0.0 (ns)	-0.2 (1.0), 0.7 (**)
GTV D90/D10%	4.1 (2.5), 6.5 (**)	15.5 (9.2), 23.7 (**)	-0.3 (0.9), 0.4 (*)	-0.4 (1.2), 1.5 (***)
Lattice pattern	1 cm spheres			
Motion direction	SI		AP	
Motion amplitude (cm)	0.5	1	0.5	1
VTVH 80%	−4.8 (1.4), −3.4 (***)	−15.8 (1.8), −13.1 (****)	-2.9 (1.3), -2.0 (****)	−9.4 (1.3), −7.6 (****)
VTVH Average	−3.9 (1.1), −3.0 (**)	−14.0 (1.7), −12.0 (***)	−3.0 (1.2), −2.1 (***)	−9.8 (1.6), −8.1 (***)
VTVL Average	−0.7 (1.0), −0.3 (ns)	−4.0 (2.6), −2.3 (*)	3.8 (0.7), 3.5 (ns)	14.3 (1.6), 13.8 (ns)
VTVL D5%	−0.4 (2.2), −0.9 (ns)	−3.6 (2.9), −1.4 (*)	4.3 (2.0), 2.9 (***)	15.8 (2.7), 13.4 (**)
VTVH/VTVL D80%	−4.3 (1.8), −3.7 (ns)	−12.8 (3.0), −12.5 (ns)	−6.0 (1.5), −5.6 (ns)	−19.8 (1.7), −20.1 (ns)
VTVH/VTVL D90%	−4.6 (2.5), −3.3 (*)	−12.9 (3.0), −9.4 (***)	-5.9 (2.4), −5.4 (ns)	−17.5 (3.0), −18.4 (ns)
VTVH/VTVL D5/D95%	−2.5 (1.2), −2.0 (ns)	−8.6 (2.8), −7.6 (ns)	−5.6 (1.2), −5.3 (ns)	−18.5 (2.6), −19.8 (ns)
VTVH/SISparing D5/D95%	−4.7 (1.0), −4.4 (ns)	−19.0 (1.9), −19.2 (ns)	−3.4 (1.3), −2.8 (ns)	−11.8 (3.3), −10.1 (*)
VTVH/VTVL D95%	−5.3 (3.7), −4.0 (ns)	−13.0 (3.5), −12.0 (ns)	−5.8 (3.5), −5.0 (ns)	−17.1 (3.5), −18.7 (ns)
GTV D5%	−3.7 (0.5), −3.4 (ns)	−10.9 (1.4), −10.4 (ns)	−0.5 (0.4), −0.6 (ns)	−1.5 (0.8), −2.4 (***)
GTV D10%	−2.8 (0.4), −2.1 (****)	–8.0 (1.2), –5.7 (****)	–0.1 (0.3), –0.1 (ns)	0.1 (0.5), 0.1 (ns)
GTV D20%	–0.9 (0.6), –0.7 (ns)	–3.0 (1.4), –2.7 (ns)	0.3 (0.2), 0.1 (*)	1.3 (0.4), 0.6 (****)
GTV D50%	4.8 (1.4), 3.4 (**)	15.6 (4.3), 10.5 (***)	0.6 (0.4), 0.6 (ns)	1.4 (0.6), 2.3 (***)
GTV D90%	2.5 (1.2), 3.1 (*)	8.3 (4.7), 13.5 (***)	0.1 (0.2), 0.0 (ns)	0.2 (0.4), 1.0 (***)
GTV D90/D10%	5.0 (1.9), 5.4 (ns)	17.0 (7.3), 20.4 (ns)	0.1 (0.4), 0.1 (ns)	0.0 (0.8), 0.9 (***)

Values for the same conditions from the phantom study are listed after a comma, again as percent differences from no motion. *p*-value magnitude (****p < 0.0001, ***p < 0.001, **p < 0.01, *p < 0.05, ns *p* > 0.05) comparing phantom value to patient values is included in parentheses after phantom values. Larger *p*-values indicate better agreement between phantom and patient values. Bolded values indicate the phantom and average patient percent differences from no motion differed by more than 5 percentage points.

**Figure 6:**
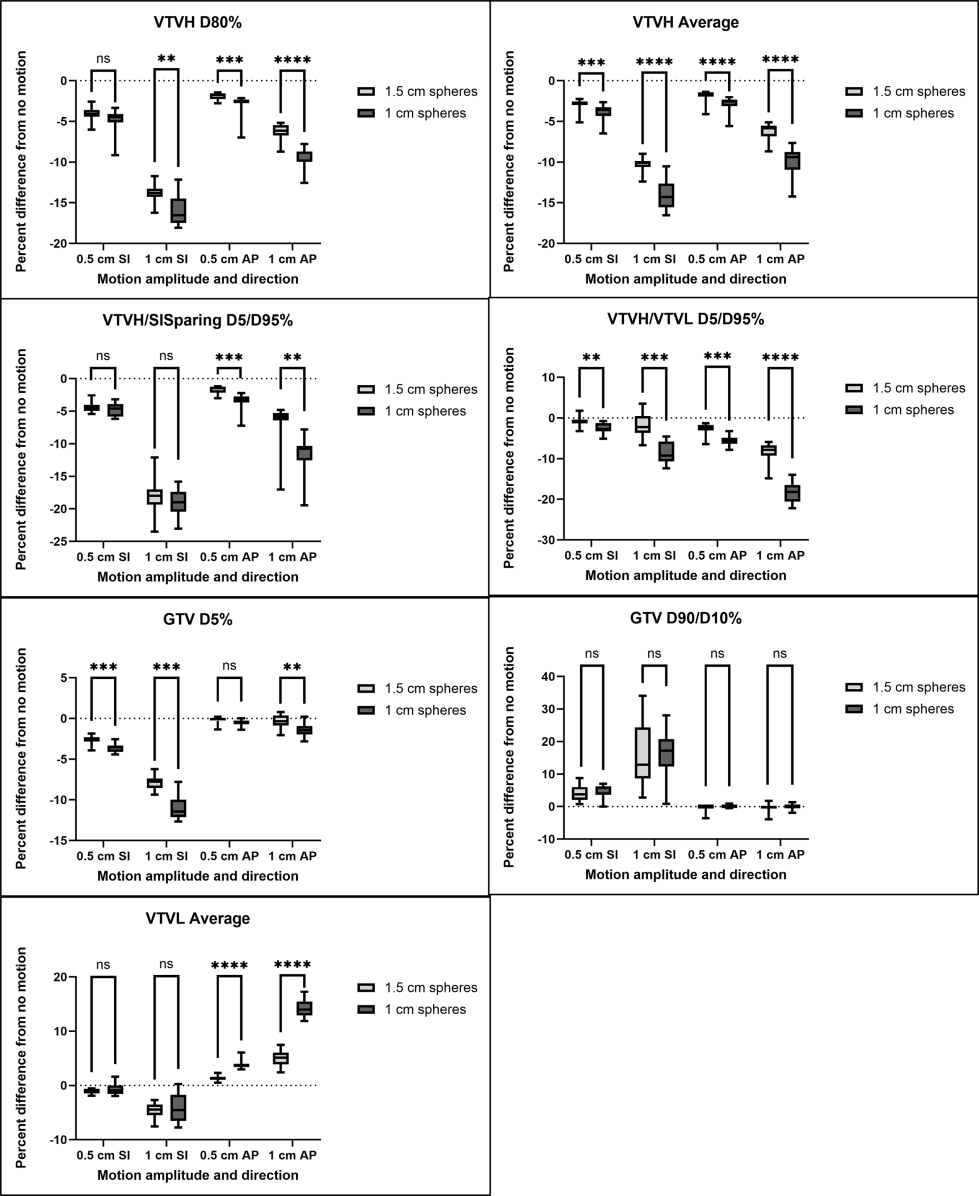
Patient anatomical variation results for 0.5 and 1 cm perturbations in the SI and AP directions; 1 cm sphere plans are shown in dark gray and 1.5 cm spheres in light gray. Results are shown as a percent difference from no motion. Statistical significance (paired Wilcoxon signed-rank test) is shown with ****p < 0.0001, ***p < 0.001, **p < 0.01, *p < 0.05, ns *p* > 0.05.

**Figure 7:**
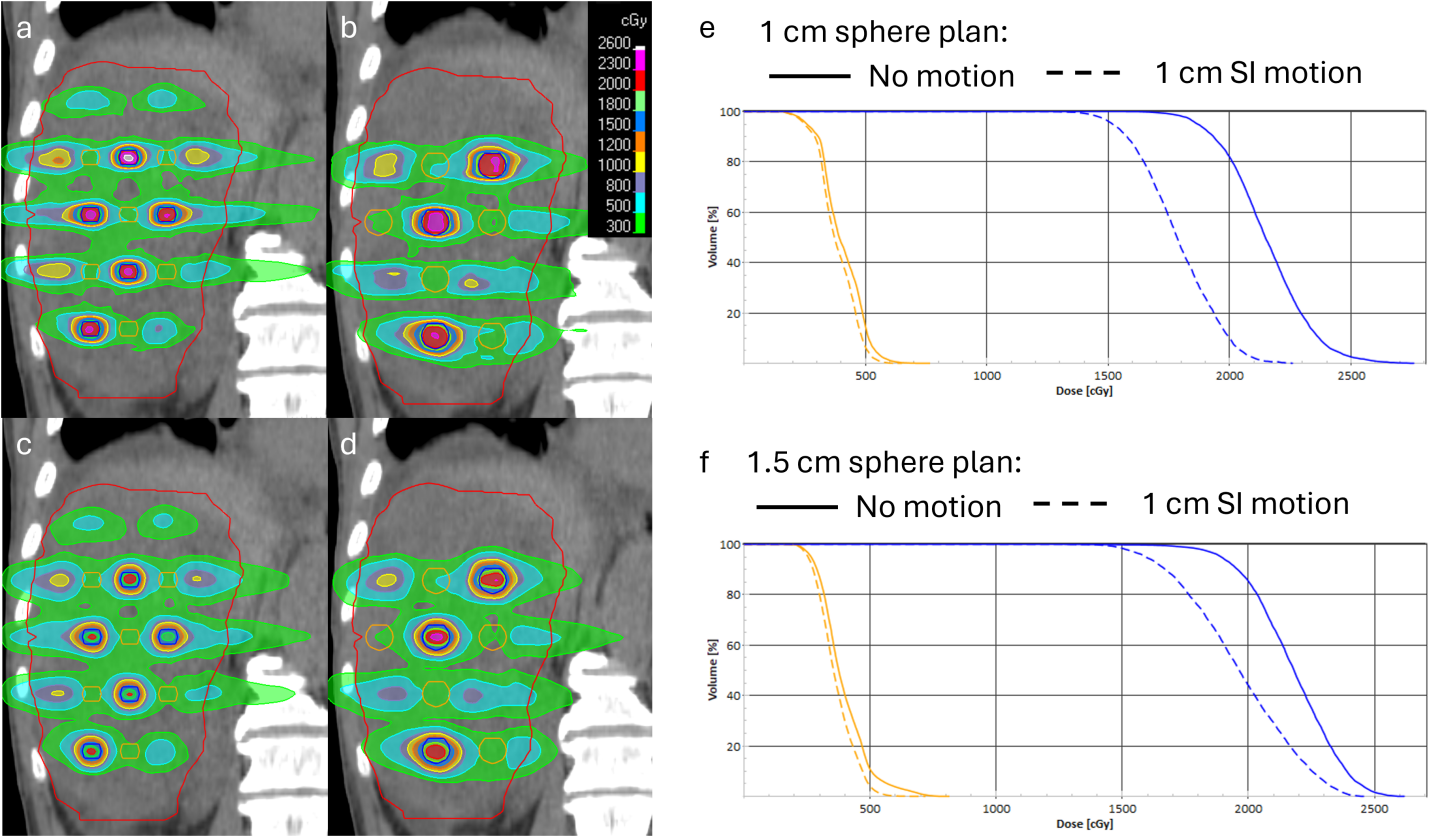
Lattice plans with 1 cm **(A)** and 1.5 cm **(B)** spheres for patient #2; 1 cm of superior–inferior motion simulated for the 1-cm **(C)** and 1.5-cm **(D)** sphere plans. DVHs for the 1-cm **(E)** and 1.5-cm **(F)** plans with original plans with solid lines and motion-blurred plans with dashed lines (VTVH dose in blue, VTVL dose in orange). A larger change in VTVH coverage is noted for the 1-cm sphere plan.

Key results from the patient anatomical variation study are summarized in **Figure 6** with additional metrics shown in **Supplementary Figure 3**. Statistical significance (*p* < 0.05) between 1 and 1.5 cm sphere lattice patterns is indicated with stars (****p < 0.0001, ***p < 0.001, **p < 0.01, *p < 0.05) and no significance with “ns.” For small SI motion, there was no significant difference in any dose parameter other than the VTVH average dose. For 1 cm SI motion and for all AP motions, there was a significant difference between 1 and 1.5 cm sphere lattice patterns in all VTVH and VTVL-related dose metrics except VTVL average and D5% doses for 1 cm SI motion. An example patient is shown in **Figure 7**, which illustrates the larger change in 1 cm compared to 1.5 cm sphere plans with 1 cm SI motion. GTV-related metrics had mixed results regarding the significance between 1 and 1.5 cm sphere plans.

### 3.3 Target deformation analysis

Unlike the phantom and anatomical variation study, there were generally no significant differences between 1 and 1.5 cm sphere plans for either the deformed or perturbed plans, except for the VTVH dose metrics. *p*-values are summarized in **Table 3**. Motion between breath-hold images was generally small with a few exceptions, which likely contributed to the lack of significant difference.

**Table 3:** *p*-values (paired Wilcoxon signed-rank test) for deformed and perturbed plans comparing 1 and 1.5 cm and deformed and perturbed.

	1 cm vs. 1.5 cm spheres		Perturbed vs. deformed	
	Deformed	Perturbed	1 cm spheres	1.5 cm spheres
VTVH 80%	**0.01 (−2.3)**	0.06 (−1.9)	0.9 (0.3)	0.43 (−0.4)
VTVH average	**0.05 (−1.2)**	**0.02 (−1.3)**	0.51 (−0.0)	0.09 (−0.6)
VTVL average	0.08 (1.1)	0.08 (1.3)	0.22 (−0.4)	**0.03 (−0.6)**
VTVL D5%	0.62 (1.5)	0.37 (0.6)	0.22 (−0.7)	**0.01 (−0.4)**
VTVH/VTVL D80%	0.09 (−3.1)	**0.01 (−3.8)**	0.5 (0.8)	0.67 (-0.2)
VTVH/VTVL D90%	0.06 (−3.6)	0.16 (−2.2)	0.9 (0.2)	0.17 (−0.5)
VTVH/VTVL D95%	0.12 (−3.0)	0.62 (−0.4)	>0.99 (0.1)	0.07 (−2.0)
VTVH/VTVL D5/D95%	0.08 (−1.7)	**0.003 (−4.2)**	0.76 (−0.1)	0.99 (−0.08)
VTVH/SISparing D5/D95%	0.19 (−1.3)	0.09 (−1.7)	0.08 (−1.6)	0.3 (−1.3)
GTV D5%	0.19 (−0.5)	0.5 (−1.2)	0.62 (−0.1)	0.13 (−0.4)
GTV D10%	**0.004 (−0.7)**	**0.006 (−0.4)**	0.61 (0.1)	0.3 (0.0)
GTV D20%	0.19 (0.4)	**0.002 (0.5)**	0.96 (0.0)	0.42 (−0.2)
GTV D50%	0.67 (−0.4)	0.58 (−0.5)	0.35 (0.5)	0.17 (0.1)
GTV D90%	0.24 (−0.9)	0.57 (−0.2)	0.68 (0.0)	0.68 (0.6)
GTV D90/D10	>0.99 (0.0)	0.87 (0.3)	0.17 (2.3)	0.26 (3.0)

Significant values are in bold. Median of the differences is in parentheses.

**Figure 8:**
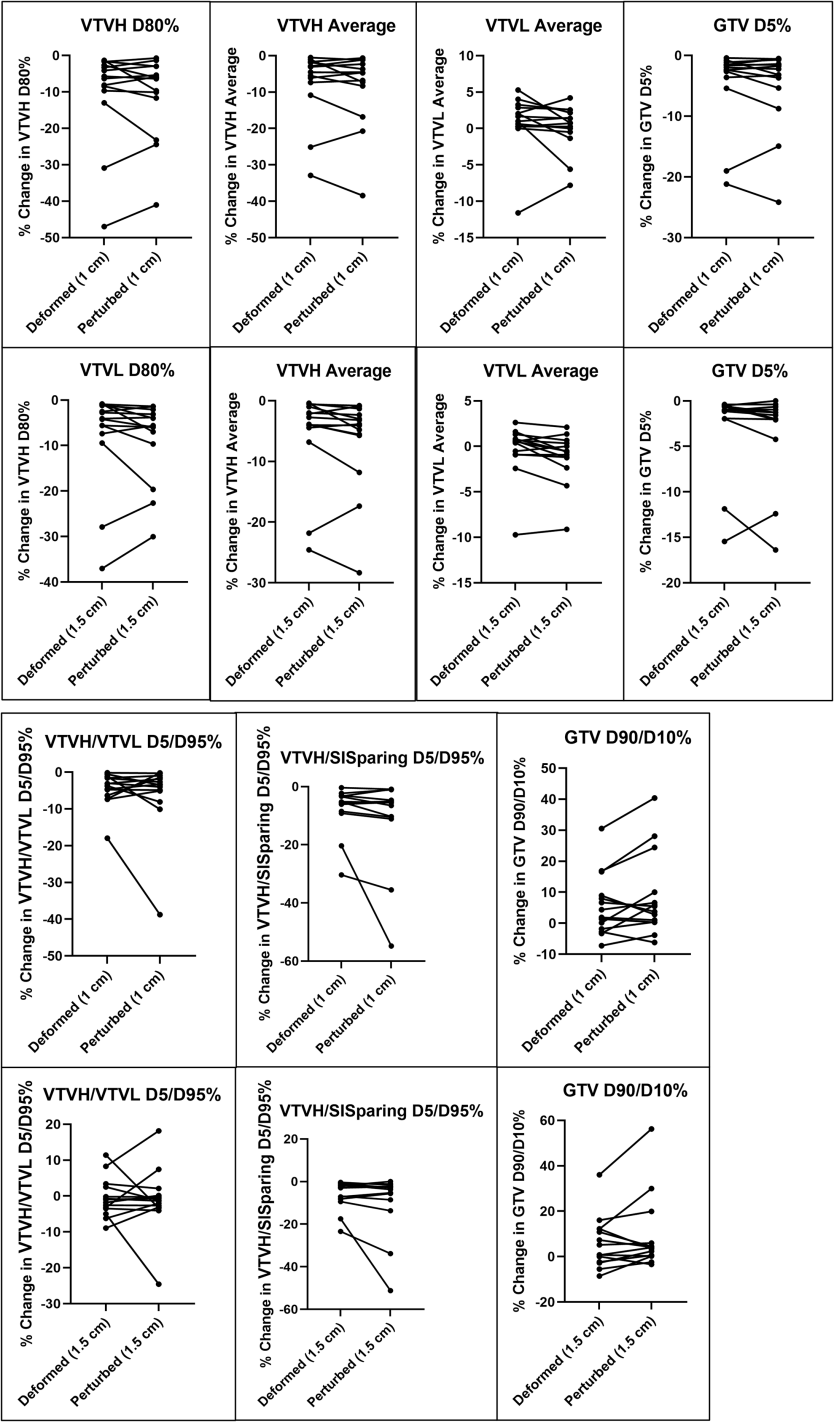
Comparison between deformed and perturbed plans for different lattice-related dose metrics. Each patient is represented by connected dots for deformed and perturbed plans to demonstrate where these techniques have the largest changes; 1 cm spheres are shown in the top row and 1.5 cm in the bottom row.

**Figure 9:**
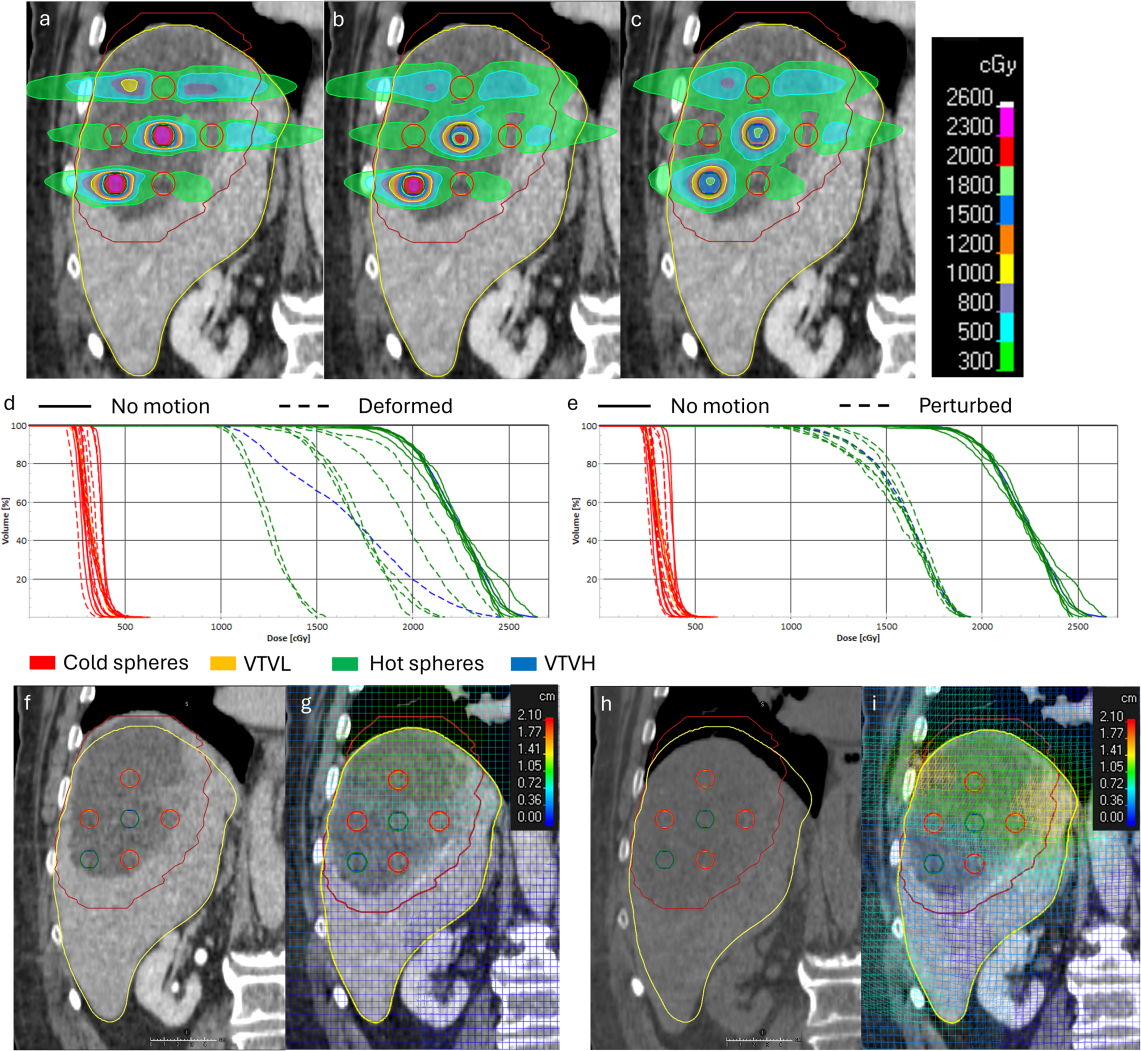
Example of a patient (#10) with a large tumor motion and deformation. Maximum rigid tumor motion was 0.6 (RL), 1.8 (SI), and 1.1 (AP) cm. The original plan is shown in **(A)**, the deformed plan in **(B)**, and the perturbed plan in **(C)**. The liver contour is displayed in yellow and the GTV in red. DVHs for deformed (dashed) compared to no motion plan (solid) are shown in **(D)**, with a similar perturbed DVH comparison in **(E)**. One visible hot sphere with low coverage and one with nearly full coverage are seen in the deformed dose plan coronal slice. The spread-out hot sphere DVHs illustrate this effect. The hot sphere coverage is somewhat low in all visible spheres in the perturbed dose plan as illustrated by the clustered hot spheres in the perturbed DVH. Large deformation of the liver near the GTV is thought to be the cause of the differences between deformed and perturbed plans in this case. Two BH images are displayed in **(F)** and **(H)** with contours from the planning CT overlaid. Deformation grids are displayed over deformed BH images in **(G)** for the BH image in **(F)** and in **(I)** for the BH image in **(H)**. For context, the percent change in VTVH D80% was 10.8% and 8.9% for the deformed and perturbed plans, respectively; VTVL average was similarly 3.0% and 2.8% and VTVH/VTVL D5/D95% 5.5% and 8.9%.

Generally, there was no significant difference between perturbed and deformed plans for the same patient and plan. The exception is with the VTVL dose metrics for 1.5 cm spheres. Overall, *p*-values were lower for 1.5 cm plans than for 1 cm plans. **Figure 8** demonstrates the difference in dose metrics between the deformed and perturbed versions of the same plan, with connected dots representing the same patient and original plan. **Supplementary Figure 4** shows similar results for more dose metrics. Generally, deformed and perturbed plans had similar results, especially for small motion as indicated by the cluster of nearly horizontal lines near zero percent change from no motion. However, there were notable outliers, particularly for patients with larger motion overall. An example of one such outlier is shown in **Figure 9**, where the shapes of the VTVH DVH between the deformed and perturbed plan are noticeably different. This patient had a large deformation in the liver shape near the GTV as illustrated in **Figures 9F–H**. The deformation resulted in some spheres being strongly impacted by motion and others not in the deformed plan as illustrated by the large spread in individual sphere DVHs. The perturbed plan naturally has a similar response for all spheres, which results in a sharper DVH curve for VTVH and more clustered DVHs for individual spheres.

## 4 Discussion

In this work, the effect of respiratory motion on lattice dose distributions was systematically studied in a uniform phantom for two common lattice patterns and verified in a patient dataset. Relevant lattice dose parameters remained relatively stable up to approximately 0.5 cm of motion for 1 cm sphere plans and close to 1 cm of motion for 1.5 cm sphere plans. Based on these findings and in an effort to balance feasibility with high-quality dose distributions, the loose cutoffs of 0.5 cm of tumor motion for 1 cm sphere plans and 1.0 cm for 1.5 sphere plans are proposed. Above these cutoffs, motion-limiting strategies such as breath hold, compression belt, or free breathing gating are recommended when clinically feasible. If the valley dose proves to be of primary importance in LRT over PVDR, slightly larger SI (but not AP) motion could be tolerated, as VTVL metrics showed limited changes for motion in the SI direction. Using these cutoffs will allow clinicians to confidently treat patients with shallow respiratory motion without creating undue burden on the patient and to achieve high-quality dose distributions in patients with larger breathing motion. Additionally, adoption of standard cutoffs can improve the interpretability of retrospective outcome analysis and clinical trial data, leading to a more accurate understanding of the efficacy of LRT for thoracic and abdominal tumors. These cutoffs are similar to those proposed without explicit evidence in Grams et al. (28), which suggests a 0.5-cm cutoff with a low bar for allowing larger motion for clinical reasons. For lung stereotactic body radiation therapy (SBRT) with FFF beams, Fernandez et al. (29) showed satisfactory PTV coverage for 1 cm but not 2 cm of motion in their moving phantom study. While the dose distribution, target definition, and clinical goals are different with SFRT relative to SBRT, it suggests that our cutoffs are similar to general motion management practice. Future work should include phantom measurements to validate these cutoffs.

The decision to separate recommendations based on sphere size was motivated by a consistent difference observed in the sensitivity of LRT-related dose metrics to motion between 1 and 1.5 cm sphere plans. In general, 1 cm sphere plans showed a higher sensitivity to motion than 1.5 cm sphere plans as evidenced by trends in the phantom analysis and statistical differences in the patient anatomical variation analysis. These differences are likely due to the sharper dose distributions needed to achieve coverage of the smaller spheres with tighter spacing. However, this sharper dose fall-off also results in somewhat higher PVDR to begin with, which should be taken into consideration when choosing a treatment strategy. These results are consistent with the findings from Naqvi et al. (25) who showed a larger dose degradation for small GRID hole diameter and for tighter spacing. They generally reported dose degradation with smaller respiratory motion than reported in this study. The reason for this discrepancy is likely tied in part to the tighter spacing between peaks used in that study (the largest of 2.1 cm in their analysis compared to the smallest of 2.8 cm in this analysis). Taken together with Naqvi’s work, these results suggest that both sphere size and spacing play a role in the sensitivity to motion.

Generally, SI motion had a stronger effect on coverage of hot dose spheres and overall PVDR, but AP motion had a stronger effect on low-dose spheres. The directional dependence on the dose metrics as seen in **Figure 4** can be understood by thinking through what portions of the dose distribution are being averaged together with motion. One important feature of the dose distribution is that the dose between each SI plane is lower than the dose to the cold spheres, since the entrance and exit dose from the hot spheres often passes through these cold spheres. Relatedly, dose falls off faster in the SI direction than the AP direction. SI motion tends to decrease the dose to both hot and cold spheres, since the low dose between planes is blurred into both structures. With AP motion, hot and cold spot doses begin to mix, resulting in a lower dose to hot spheres but a higher dose to cold spheres. Due to the faster fall off in the SI direction, the dose to hot spheres decreases faster for SI compared to AP motion. Conversely, doses to cold spheres increase more rapidly for AP motion compared to SI motion due to the higher doses surrounding the cold spheres being blurred into them. Overall, for simplicity’s sake, rigid SI motion can be used when determining the tumor motion for cutoff evaluation. However, if appreciable AP or lateral motion exists, the approximate total vector motion can be used.

The above results and recommendations assumed rigid tumor motion despite the fact that the abdomen and thorax can experience large deformations. The results of the deformation analysis suggest that in general the effect of deformation can be ignored and rigid motion assumed, especially for a relatively small motion. However, some patients showed large differences in dose metrics between rigid and deformed motion due to deformation of the GTV. Patients, where noticeable deformation is observed, may benefit from further analysis using deformable image registration along the lines performed in Section 3.3 to aid in motion management strategy decisions.

These analyses used a somewhat simplified motion model in assuming sinusoidal motion and sampling the respiratory curve. While generally a good approximation, respiratory motion that differs appreciably from this pattern may have a somewhat different motion response. Other studies have used Monte Carlo modeling to accurately model a range of respiratory patterns in standard treatments (30–35). As future work, similar techniques could be applied to LRT for a fuller understanding of the effect of different breathing patterns on lattice dose distributions. Another simplification applied was ignoring the impacts from the interplay effect (29, 36–38). Given that LRT is generally delivered in one or a few fractions, the interplay effect is expected to be greater than with fractionated radiation therapy, where the effect can be washed out by different relative motions each day. The impact of the interplay effect in LRT is beyond the scope of this paper but warrants future investigation including phantom measurements to validate results. Another limitation of this study is that given the clinical practice at our institution, most patients used in the deformation analysis were treated under breath hold and were not simulated with 4DCT. The smaller variation between images in breath hold compared to 4DCT likely contributed to the absence of significant differences between perturbed and deformed dose distributions. In a sample of free-breathing patients, more differences between these methods may exist. In general, paying attention to large tumor deformations, whether free breathing or breath hold, will reduce the chances for unexpected dose behavior. For the patient anatomical variation analysis, we selected a subset of phantom motion amplitudes to replicate. While patient values were generally in agreement with phantom measurements for the range of amplitudes selected, it is possible that there is greater deviation for larger amplitudes. There is a variety of valid methods for creating lattice dose distributions. Care should be exercised when directly applying the results of this study to LRT methods that vary substantially from the one described here.

## Data Availability

The raw data supporting the conclusions of this article will be made available by the authors, without undue reservation.

## Supplementary material

Supplementary Figure 1 Sample optimization parameters for 1 cm (top) and 1.5 cm (bottom) plan for the same patient. Contours are as defined in **Figure 2**. zIC, zMC, and zOC are rings around VTVH and zIL are rings around VTVL. Individual hot (green with name starting with s) or cold (red with name starting with c) spheres can be added as optimization objectives if they are under or over covered, respectively.

Supplementary Figure 2 Effect of simulated respiratory motion on lattice related dose metrics. Left graphs focus on the difference in motion response of motion direction. Right graphs focus on sphere size dependence.

Supplementary Figure 3 Patient anatomical variation results for 0.5 and 1 cm perturbations in the SI and AP directions. 1 cm sphere plans are shown in dark gray and 1.5 cm spheres in light gray. Results are shown as a percent difference from no motion. Statistical significance (paired Wilcoxon signed-rank test) is shown with**** p < 0.0001, *** p < 0.001, ** p < 0.01, * p < 0.05, ns p > 0.05.

Supplementary Figure 4 Comparison between deformed and perturbed plans for different lattice related dose metrics. Each patient is represented by connected dots for deformed and perturbed plans to demonstrate where these techniques have the largest changes. 1 cm sphere are shown in the top row and 1.5 cm in the bottom row.
