# Supplementary figures and images for: Respiratory motion effects and plan robustness for lattice radiation therapy
Source: Front Oncol. 2026 Feb 25;16:1731981. doi: 10.3389/fonc.2026.1731981 (PMC12975452; doi:10.3389/fonc.2026.1731981)

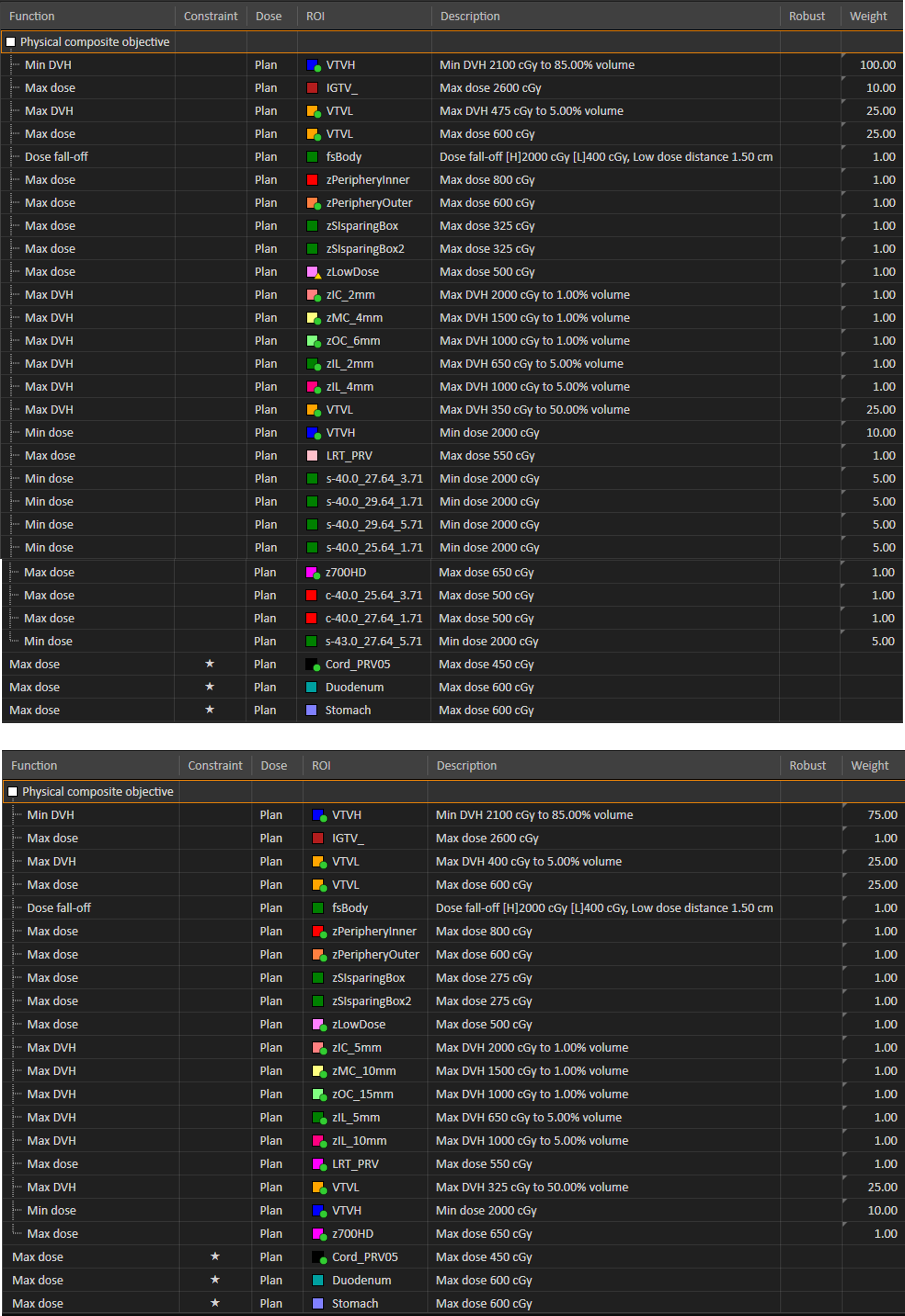

Supplement: Supplementary Figure 1 — Sample optimization parameters for 1 cm (top) and 1.5 cm (bottom) plan for the same patient. Contours are as defined in Figure 2. zIC, zMC, and zOC are rings around VTVH and zIL are rings around VTVL. Individual hot (green with name starting with s) or cold (red with name starting with c) spheres can be added as optimization objectives if they are under or over covered, respectively. [file Image1.tif]

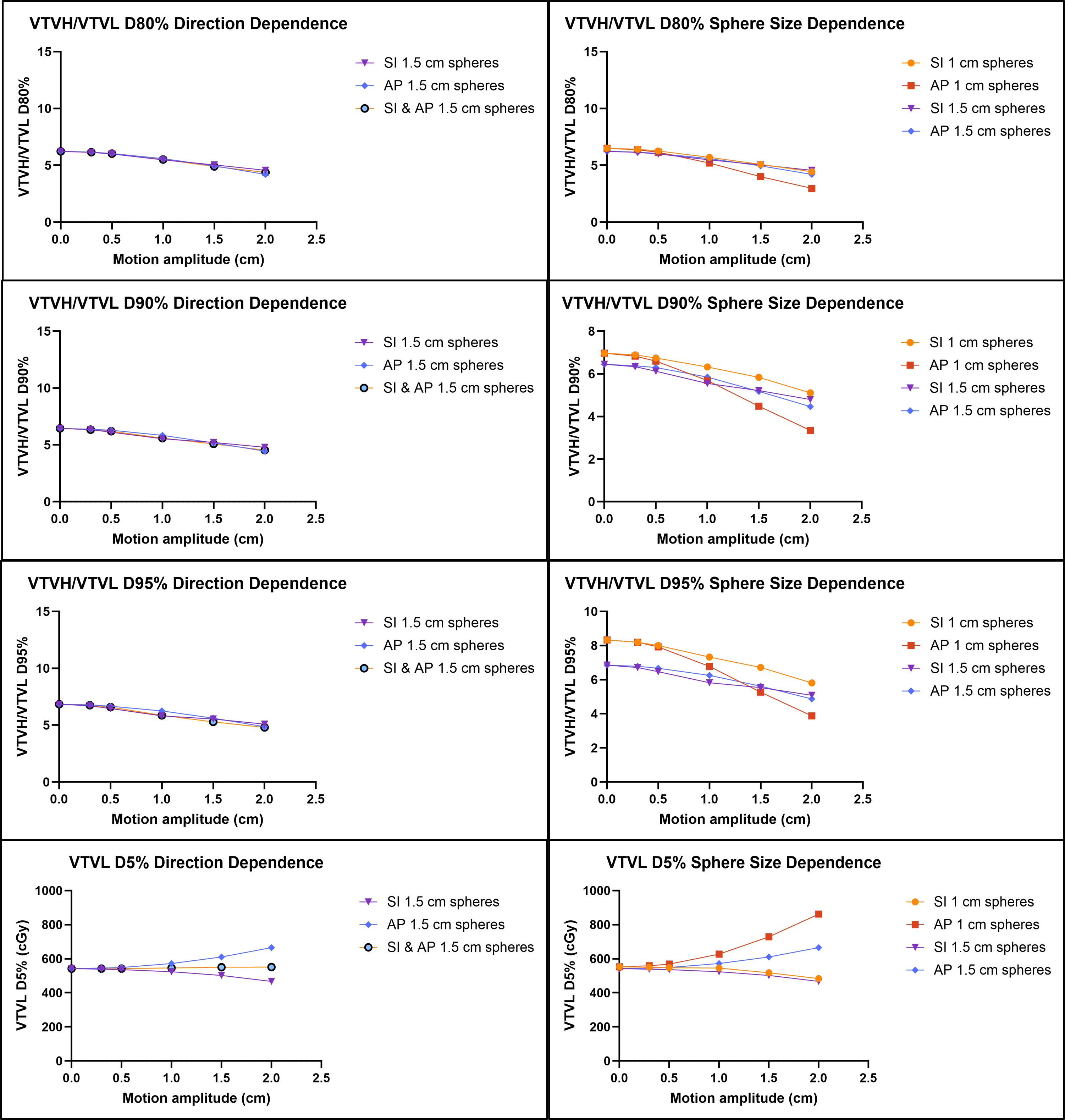

Supplement: Supplementary Figure 2 — Effect of simulated respiratory motion on lattice related dose metrics. Left graphs focus on the difference in motion response of motion direction. Right graphs focus on sphere size dependence. [file Image2.tif]

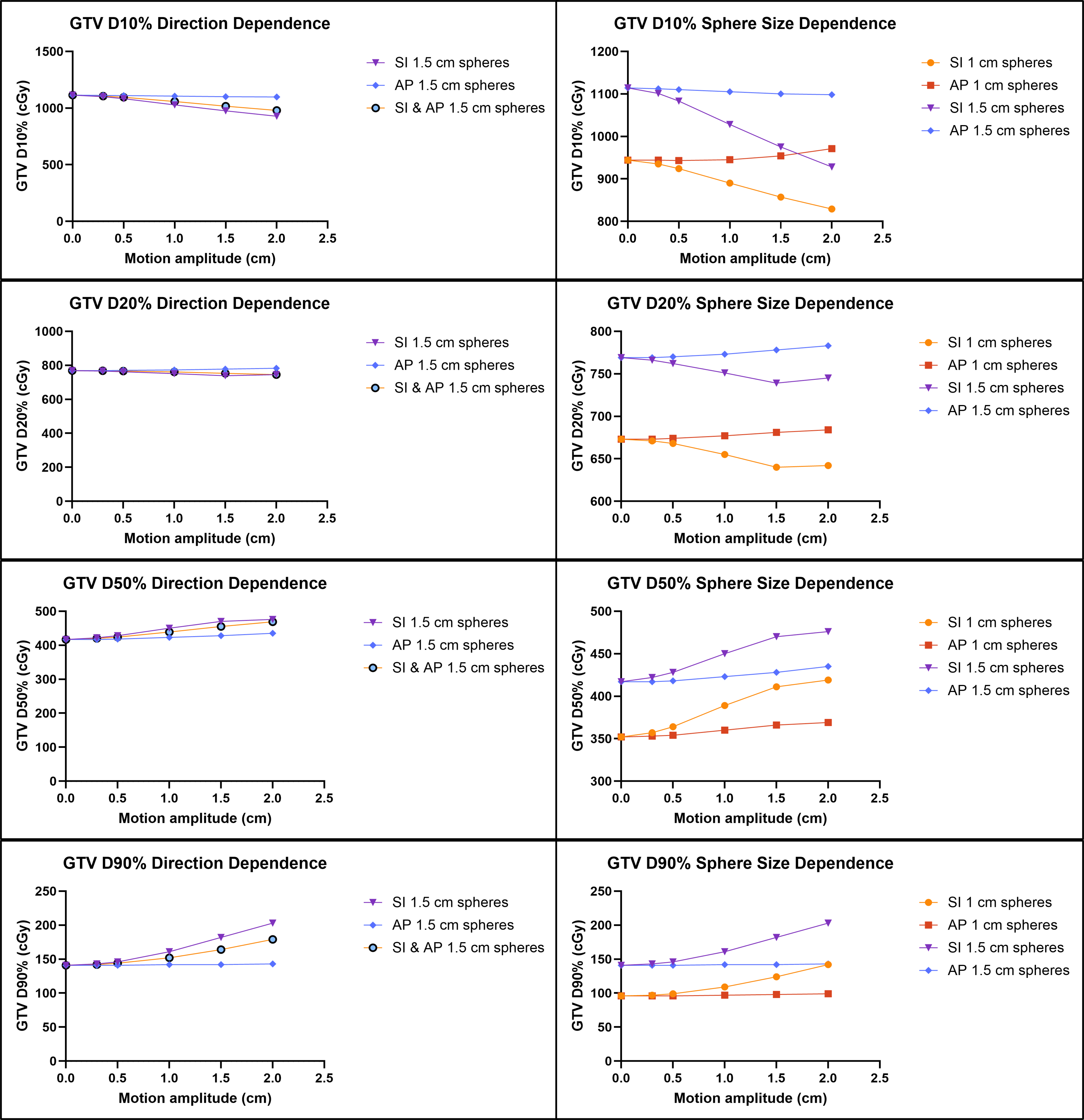

Supplement: Supplementary Figure 3 — Patient anatomical variation results for 0.5 and 1 cm perturbations in the SI and AP directions. 1 cm sphere plans are shown in dark gray and 1.5 cm spheres in light gray. Results are shown as a percent difference from no motion. Statistical significance (paired Wilcoxon signed-rank test) is shown with**** p < 0.0001, *** p < 0.001, ** p < 0.01, * p < 0.05, ns p > 0.05. [file Image3.tif]

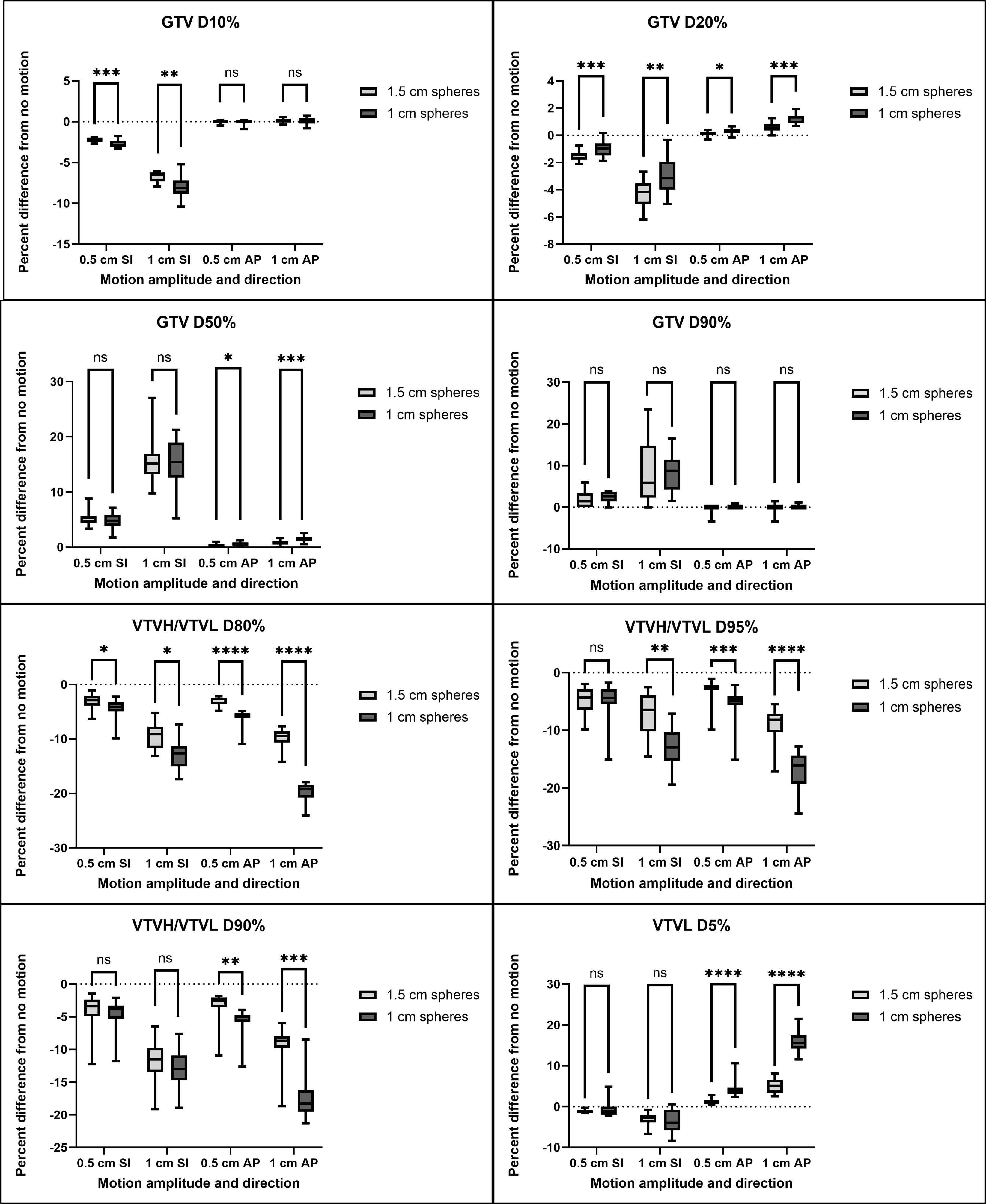

Supplement: Supplementary Figure 4 — Comparison between deformed and perturbed plans for different lattice related dose metrics. Each patient is represented by connected dots for deformed and perturbed plans to demonstrate where these techniques have the largest changes. 1 cm sphere are shown in the top row and 1.5 cm in the bottom row. [file Image4.tif]

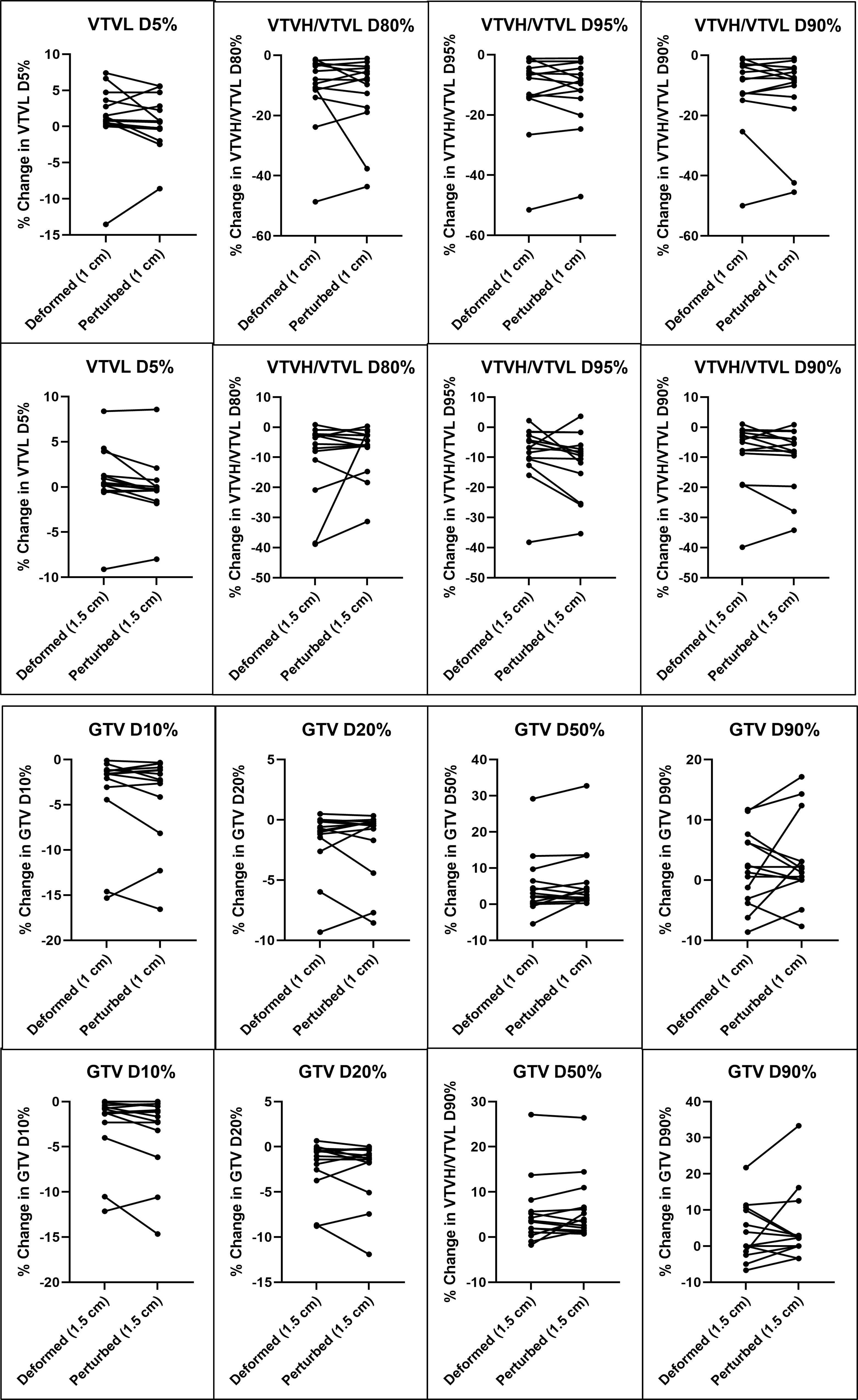

Supplement: Supplementary file 5 [file Image5.tif]
